# Bacteriophage lysins as antibacterials

**DOI:** 10.1186/s13054-017-1681-6

**Published:** 2017-05-03

**Authors:** Umender Sharma, Vivek D. Paul

**Affiliations:** GangaGen Biotechnologies Pvt Ltd, Yeshwantpur, Bangalore, India

We read the editorial entitled “Non-antibiotic treatments for bacterial diseases in an era of progressive antibiotic resistance” by Steven M. Opal published in *Critical Care* [1]. We want to congratulate the author for this timely editorial and appreciate the efforts to highlight various non-antibiotic-based approaches being pursued for treating drug-resistant bacteria. We fully agree that there is a need to consider these approaches as serious alternatives to conventional antibiotics.

In the editorial, six possible options have been listed as non-antibiotic inhibitors of bacterial growth. We agree that these are viable options which include devices and chemical or biological compounds for addressing the problem of bacterial drug-resistance. Although we understand that this is not an exhaustive list of possible options, we would like to suggest that bacteriophage “lysins”, including both endolysins (which help the phage in releasing the intracellular virus particles by cleaving the peptidoglycan from inside) and ectolysins (which help the phage in injecting DNA into the bacterial cell by cleaving the peptidoglycan on the external surface) can also be considered as alternative options for treating drug-resistant bacteria. When added to a bacterial culture in broth or buffer, the lysins kill bacteria rapidly by cleaving peptidoglycan, a cell wall component specific to bacteria. Because of their specific nature, lysins kill only the target bacteria and thus do not alter the microbiome. Lysins can be easily produced in *Escherichia coli* as recombinant proteins and properties such as rapid killing, specificity, ability to kill antibiotic persisters, low rates of resistance and profound antibiofilm activity [2–4] make them good candidates for clinical development for treating serious infections. Several lysins such as CF-301, N-Rephasin, P128 and Art-175 are in various stages of advanced pre-clinical or clinical development as antibacterials for treating infections caused by drug-resistant Gram-positive and Gram-negative pathogens [3–5]. Antibacterial efficacy of a number of lysins has been demonstrated in relevant animal models and it has also been observed that the presence of anti-lysin antibodies does not impact the lysin efficacy in vivo [6]. Thus, keeping in mind the recent advances made in the area of discovery and clinical development of phage lysins to combat drug-resistant bacteria, we propose that lysins should also be highlighted as alternative options under the “non-antibiotic” approaches.

## Abbreviations

MDR: Multiple antibiotic drug-resistant
