# Supplementary material for: Bacteriophage lysins as antibacterials
Source: Crit Care. 2017 May 3;21:99. doi: 10.1186/s13054-017-1681-6 (PMC5415796; doi:10.1186/s13054-017-1681-6)
Supplement: Related texts

# Reply: Author’s response

**Authors:** Steven M. Opal

I heartily agree with the comments made by Sharma and Paul regarding the potential contribution of phage-medicated endolysin as a novel therapeutic option against multiple antibiotic drug-resistant (MDR) bacterial pathogens. Phage employ a two-component enzyme system, known as holin and endolysin, to allow newly generated phage progeny to exit the dying bacterial cell and invade other susceptible host cells. Holin is a bacterial cell membrane permeablizing enzyme that creates pores within the inner membrane of bacteria, while endolysin destroys the cross-linking of the peptidoglycan layer that constitutes the outer membrane. Holin sets the pace of lysis to correspond to the maximum rate of phage synthesis just before the parasitized bacterial cell dies. At that moment endolysin floods the outer membrane, thereby lysing the cell allowing the new generation of phage particles to be released into the environment [7].

Recombinant forms of lysins have been synthesized and are now being used therapeutically in experimental models of bacterial infection [3, 7–9]. Their protective activity is impressive with clearance rates of bacterial pathogens that rival standard antibiotics. This antibiotic-independent, lytic enzyme should be fully active against MDR pathogens [3, 7–9]. If clinical testing can assure safety and efficacy of this highly potent antibacterial peptide, lysin therapy might become a welcome addition to our dwindling options to treat MDR pathogens.
